# Experiment-guided tuning of muscle–tendon parameters to estimate muscle fiber lengths and passive forces

**DOI:** 10.1038/s41598-024-65183-1

**Published:** 2024-06-25

**Authors:** Israel Luis, Maarten Afschrift, Elena M. Gutierrez-Farewik

**Affiliations:** 1 KTH MoveAbility, Department Engineering Mechanics, KTH Royal Institute of Technology, Osquars Backe 18, Plan 4, 11428 Stockholm, Sweden; 2 Faculty of Behavioural and Movement Sciences, VU Amsterdam, Amsterdam, The Netherlands; 3 Department of Women’s and Children’s Health, Karolinska Institutet, Stockholm, Sweden

**Keywords:** Muscle fiber length, Musculoskeletal parameter, Muscle–tendon mechanics, Scaling method, Metabolic energy cost, Biomedical engineering, Mechanical engineering

## Abstract

The workflow to simulate motion with recorded data usually starts with selecting a generic musculoskeletal model and scaling it to represent subject-specific characteristics. Simulating muscle dynamics with muscle–tendon parameters computed from existing scaling methods in literature, however, yields some inconsistencies compared to measurable outcomes. For instance, simulating fiber lengths and muscle excitations during walking with linearly scaled parameters does not resemble established patterns in the literature. This study presents a tool that leverages reported in vivo experimental observations to tune muscle–tendon parameters and evaluates their influence in estimating muscle excitations and metabolic costs during walking. From a scaled generic musculoskeletal model, we tuned optimal fiber length, tendon slack length, and tendon stiffness to match reported fiber lengths from ultrasound imaging and muscle passive force–length relationships to match reported in vivo joint moment–angle relationships. With tuned parameters, muscle contracted more isometrically, and soleus’s operating range was better estimated than with linearly scaled parameters. Also, with tuned parameters, on/off timing of nearly all muscles’ excitations in the model agreed with reported electromyographic signals, and metabolic rate trajectories varied significantly throughout the gait cycle compared to linearly scaled parameters. Our tool, freely available online, can customize muscle–tendon parameters easily and be adapted to incorporate more experimental data.

## Introduction

Musculoskeletal simulations have provided insights into muscle, tendon, and joint mechanics^1^. The simulated multibody mechanics: joint angles and moments can be informed by recorded motion data to estimate muscle–tendon mechanics. A workflow to simulate motion with recorded motion data starts with creating a musculoskeletal model^2^ or, more commonly, selecting one from the literature. If a generic musculoskeletal model is selected, the properties of such a model need to be adjusted to present subject characteristics, for instance, body segments’ dimensions or muscle–tendon parameters. The computed muscle–tendon parameter can greatly influence the estimated muscle–tendon mechanics. Sensitivity analyses have shown that optimal fiber length, tendon slack length, and, to a lesser extent, maximum isometric force are the primary parameters influencing the predicted muscle forces^3–6^. In this regard, accurately estimating such sensitive parameters will increase confidence of predicted muscle–tendon mechanics.

Current scaling methods in literature to estimate optimal fiber lengths and tendon slack lengths, however, seem to yield some inconsistencies compared to established patterns in literature. In OpenSim software^7^, optimal fiber length and tendon slack length are determined by preserving the ratio between generic and models scaled towards the subject. Another standard method is to estimate these parameters by mapping the operating range of muscle fiber lengths during a specific task in the generic model into the scaled model^8^. In a previous study, we reported similar estimated muscle fiber lengths from both of these scaling methods in several musculoskeletal models during walking, and none uniformly agreed well with observed fiber lengths from ultrasound imaging^9^. We observed such disagreement when muscle redundancy was solved based on the minimal sum of squared muscle activations; this criterion has been reported to estimate muscle excitations better than other formulations, such as minimal metabolic rates or muscle forces when joint mechanics is prescribed^10^. Likely, a more suitable method to estimate muscle–tendon parameters enables a better representation of fiber lengths.

Scaling muscle passive force–length relationships is often overlooked despite playing a significant role in estimating muscle–tendon force, particularly around the knee and hip joint. During scaling, the muscle passive force–length relationships are the same among all muscles as in the generic model, whereas experimental studies have shown variation among them^11,12^. Incorporating such variations would better represent muscles’ passive force and thus improve our understanding of the role of active and passive force generation^13^ and energetics^14^ during motion. Despite this importance, methods to ensure that passive moment–angle relationships in scaled musculoskeletal models are consistent with prior findings in the literature are scarce.

Methods for identifying muscle–tendon parameters from recorded muscle data are time- and resource-intensive. Calibration based on recorded muscle data, such as electromyographic signals (EMGs)^15–17^ ultrasound imaging or both^18–21^ enable the computation of muscle forces accounting for subject-specific characteristics. This approach might be particularly relevant in a clinical population where muscle architecture is known to vary substantially among participants^20^. Yet, it comes with the expense of lengthy data collection and post-processing; muscle–tendon parameter identification requires extensive calibration over a broad range of tasks^17,22^. Also, despite the obvious advantages of model individualization, studies focused on investigating patterns such as describing muscle force profiles during activities like pedaling, running, and walking^13^ or the effect of ideal devices to reduce metabolic rates during gait^23^ would unlikely adopt such a time- and resource-intensive approach unless the research question addresses subject-specific characterization.

Tuning muscle–tendon properties based on reported experimental observations can overcome the limitations of existing scaling methods and better represent muscle–tendon mechanics. By directly integrating in vivo observations from established patterns in literature to tune muscle–tendon parameters^24^, simulated muscle fiber lengths and passive moment–angle relationships can closely mimic experimental data, thus potentially increasing the confidence of other estimated quantities such as muscle excitations or metabolic energy cost. This study presents a method that leverages reported experimental findings: fiber lengths and passive moment–angle relationships to tune muscle–tendon parameters in scaled musculoskeletal models. We simulated walking without and with tuned parameters and evaluated the agreement between estimated muscle excitations and metabolic rates with reported EMGs from the literature and recorded metabolic energy costs, respectively. We hypothesized that tuning parameters significantly improve estimations of muscle excitations and metabolic rates compared to a standard method in the literature.

## Results

We introduced and evaluated a computational tool to tune muscle–tendon parameters based on prior in vivo experimental findings from the literature: fiber lengths from ultrasound imaging and passive moment–angle relationships at various ankle, knee, and hip joint angles. For each of the twelve subjects included in our study, muscle dynamics during walking were simulated with three different workflows:

- LIN: The conventional linear scaling workflow in OpenSim.
- FIB: With tuned optimal fiber length, tendon slack lengths, and tendon compliance in soleus, gastrocnemius lateralis and medialis, and vastus lateralis medialis and intermedius from ultrasound imaging.
- ALL: Also including tuned muscle passive force–length relationships in all muscles from joint passive moment–angle relationships.

We described the influence of tuned parameters in computed fiber velocities and muscle forces. The estimated muscle excitations and whole-body average metabolic rates were compared to EMGs reported in the literature and our recorded metabolic measurements.

### Computed muscle *fiber* lengths during walking

**Figure 1:**
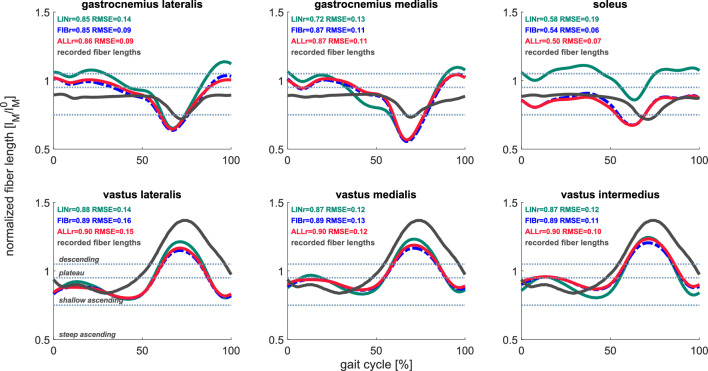
Computed and experimental fiber lengths. Average fiber lengths computed from the conventional workflow (LIN, *green*), with tuned optimal fiber length, tendon slack lengths, and tendon compliance (FIB, *dashed blue*), and including tuned muscle passive force–length relationships (ALL, *red*). Experimental muscle fiber lengths were obtained by digitalizing data reported by Farris and Raiteri^48^ and Bohm et al.^50^ (*black*). Average values are computed among all subjects.

The computed fiber lengths with tuned muscle–tendon parameters showed low to moderate differences compared to the conventional workflow. The selected non-normalized Achilles tendon stiffnesses (139.9 ± 18.5 N/mm [mean ± SD]) were lower than the tuned stiffnesses (164.7 ± 38.1 N/mm [mean ± SD]) yet not significant (p = 0.06). Tuned optimal fiber lengths were in close agreement with values reported by cadaveric^25^ and MRI^26^ studies in most muscles (Supplementary Table 1). Simulating walking with tuned optimal fiber length, tendon slack length, and tendon stiffness (FIB and ALL) improved the estimation of soleus fiber lengths, specifically its operating range, compared to the conventional workflow (LIN) (Fig. 1). The improvement in other muscles was less pronounced. Fiber velocities showed some differences with tuned optimal fiber length, tendon slack length, and tendon stiffness (FIB and ALL)—gastrocnemius fiber velocities were lower during mid- and terminal stance, and vasti fiber velocities were lower during loading response and midstance compared to the conventional workflow (LIN) (Supplementary Fig. 1). In this regard, these muscles contracted more isometrically with tuned parameters than with the conventional workflow. Tuning muscle passive force–length relationships only slightly affected estimated fiber lengths and velocities (Fig. 1 and Supplementary Fig. 1). Also, as a result of the change in muscle force-generating capacities, muscle–tendon forces varied, most notably in soleus and gastrocnemius medialis – soleus force peak was lower compared to the conventional workflow (LIN) while in gastrocnemius medialis was higher (Supplementary Fig. 2).

### Computed moment–angle relationship during passive motion

**Figure 2:**
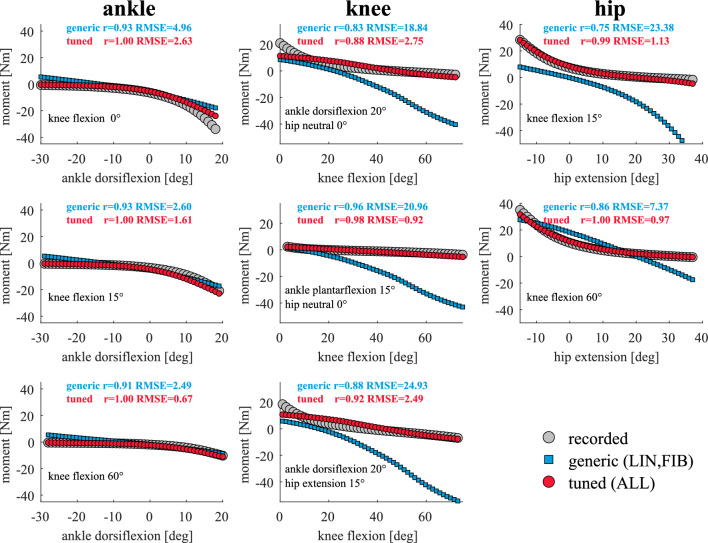
Computed and experimental joint passive moment–angle relationships. Average passive moments from in vivo measurements (*circle gray markers*) and computed with generic (*square light blue markers*) and tuned (*circle red markers*) muscle passive force–length relationships across joint ranges of motion at the ankle, knee, and hip. Experimental data was reported by Silder et al.^30^ and digitalized by Uhlrich et al. ^52^. Average values are computed among all subjects.

The computed passive moments closely resembled experimental observations. Excellent agreement (0.88 ≤ *r* ≤ 1.00 and 0.65 ≤ RMSE ≤ 2.73 Nm) was achieved by tuning muscle passive force–length relationships, except at the ankle and knee joint at large dorsiflexion (~ > 15 deg) and a nearly extended knee (Fig. 2). Tuned muscle passive force–length relationships at the knee and hip joint were mainly shifted to generate lower passive force across fiber lengths compared to generic values in most muscles except in adductor longus, biceps femoris short head, tensor fasciae latae, and gluteus minimums (Supplementary Fig. 3). At the ankle joint, soleus and gastrocnemius generated larger passive force with tuned parameters than with generic values as normalized fiber lengths increased (Supplementary Fig. 3). As a result of changes in muscle passive force-generating capacity, muscle–tendon forces varied during walking, most notably in muscles across the knee and hip joint (Supplementary Fig. 2). For instance, compared to workflows without tuned muscle passive force–length relationships (FIB and LIN), adductor magnus forces were higher at the beginning and end of the gait cycle, and vasti forces were lower during the swing phase.

### Evaluation of muscle excitations

**Figure 3:**
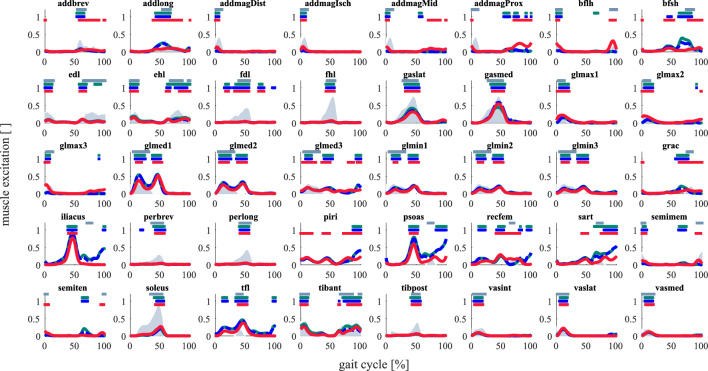
Computed and experimental muscle excitations. On/off timings are presented as horizontal lines that represent muscle excitations exceeding 50% of maximum value. Average muscle excitations computed from the conventional workflow (LIN, *green*), with tuned optimal fiber length, tendon slack lengths, and tendon compliance (FIB, *dashed blue*), and including tuned muscle passive force–length relationships (ALL, *red*). EMG signals were obtained by digitalizing data reported by Perry^59^ (*gray*). Muscle names (plot titles) refer to their abbreviations in the musculoskeletal model: adductor brevis (addbrev), adductor longus (addlong), adductor magnus (addmagDist, addmagIsch, addmagMid, and addmagProx), biceps femoris long head (bflh), biceps femoris short head (bfsh), extensor digitorum longus (edl), extensor hallucis longus (ehl), flexor digitorum longus (fdl), flexor hallucis longus (fhl), gastrocnemius lateralis (gaslat), gastrocnemius medialis (gasmed), gluteus maximus (glmax1, glmax2, and glmax3), gluteus medius (glmed1, glmed2, and glmed3), gluteus minimus (glmin1, glmin2, and glmin3), gracilis (grac), iliacus, peroneus brevis (perbrev), peroneus longus (perlong), piri, psoas, rectus femoris (recfem), sartorius (sart), semimembranosus (semimem), semitendinosus (semiten), soleus, tensor fasciae latae (tfl), tibialis anterior (tibant), tibialis posterior (tibpost), vastus intermedius (vasint), vastus lateralis (vaslat), and vastus medialis (vasmed). Average values among all subjects are illustrated.

Computed muscle excitations with tuned muscle passive force–length relationships closely resembled the on/off timing in most muscles compared to EMGs. Muscle excitations varied slightly with tuned optimal fiber length, tendon slack, and tendon stiffness (FIB) compared to the conventional workflow (LIN) (Fig. 3). Tuning of muscle passive force–length relationships (ALL) resulted in similar or improved estimation of on/off timing in all muscles except in bicep femoris short head and sartorius. The correlation of estimated muscle excitations between the conventional workflow (LIN) and tuning of optimal fiber length, tendon slack, and tendon stiffness (FIB) with EMGs in most muscles was similar ($\Delta r\approx \pm 0.2)$ or improved ($\Delta r>0.2)$ as well (Supplementary Fig. 4). Notably, correlation of muscle activation in all hamstring muscles except bicep femoris short head was substantially higher with the workflow ALL compared to the others. Overall, tuning of muscle passive force–length relationships resulted in substantially improved muscle excitation estimation of the knee flexors (e.g., bicep femoris long head) and rectus femoris and preserved good agreement in most other muscles compared to EMGs. None of our workflows captured the on/off timing of the gracilis, illiacus, or psoas excitations compared to EMGs.

### Evaluation of metabolic cost

**Figure 4:**
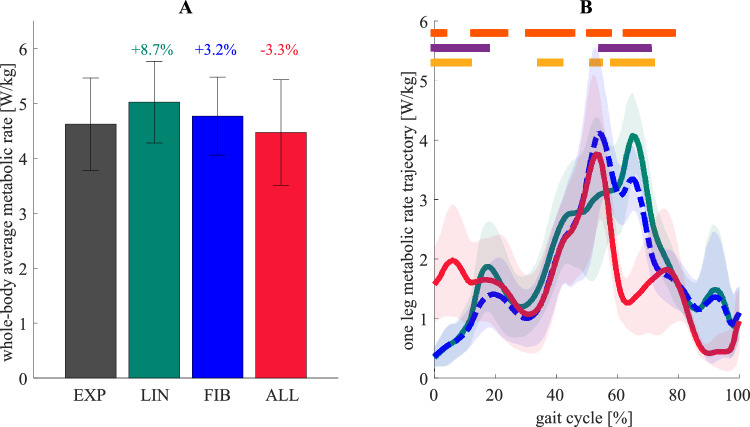
Computed and experimental metabolic rates. (**A**) Whole-body average metabolic rate and (**B**) metabolic rate in one leg computed from recorded measurements (EXP, *black*) and simulated from the conventional workflow (LIN, *green*), with tuned optimal fiber length, tendon slack lengths, and tendon compliance (FIB, *dashed blue*), and including tuned muscle passive force–length relationships (ALL, *red*) (Right). Statistically significant differences (SnPM test, p < 0.05) are shown in horizontal lines; they showed differences between workflow LIN and FIB (*orange*), workflow FIB and ALL (*purple*), and workflow LIN and ALL (*yellow*). Mean values and one standard deviation among all subjects are illustrated.

All workflows estimated whole-body average metabolic rates close to measured values, but time-series values varied significantly within gait cycle phases. The whole-body average metabolic rate in workflow LIN (5.0 ± 0.7 [mean ± SD]), FIB (4.8 ± 0.7 [mean ± SD]), and ALL (4.5 ± 1.0 [mean ± SD]) were remarkably close to measured metabolic rates (4.6 ± 0.8 [mean ± SD]) (Fig. 4-A). There was no statistically significant difference between measured metabolic rates and workflow LIN (p = 0.23), workflow FIB (p = 0.62), or workflow ALL (p = 0.51), but we found differences in the metabolic rate trajectories (${\dot{E}}_{leg}$), analyzed using statistical non-parametric mapping (SnPM) (Fig. 4-B). Compared to the conventional workflow (LIN), simulating metabolic rates during walking using tuned optimal fiber length, tendon slack length, and tendon compliance (FIB) resulted in different magnitudes across the stance phase and initial swing. Mainly, the highest metabolic rate peak differed. With the conventional workflow (LIN), the peak was estimated during the initial swing (~ 65% of the cycle (SnPM test, p < 0.05)), while with tuned parameters (FIB and ALL), during pre-swing (~ 55% of the cycle (SnPM test, p < 0.05)). This change was associated with a lower knee flexor metabolic peak during the initial swing and a higher plantarflexor metabolic peak during pre-swing (Supplementary Fig. 5). Also, compared to the conventional workflow (LIN), the metabolic rate peak of knee extensors was lower during the loading response in workflow ALL. Compared to workflow FIB, including tuned muscle passive force–length relationships (ALL) resulted in higher metabolic rates during initial contact and loading response (0–16% of the gait cycle (SnPM test, p < 0.05)) and lower during the pre-and initial swing (55–69% of the gait cycle (SnPM test, p < 0.05)). These changes were associated with higher hip extensor and hip adductor metabolic rates during initial contact and loading response and lower knee and hip flexor metabolic rates during pre- and initial swing (Supplementary Fig. 5). Overall, including all tuned parameters (ALL) resulted in somewhat lower whole-body average metabolic rates than using the conventional workflow (LIN), higher metabolic rates during early stance (0 to 11% of the gait cycle (SnPM test, p < 0.05)), earlier metabolic rate peak (~ 55% of the cycle (SnPM test, p < 0.05)), and lower metabolic rates during initial swing (59–71% of the gait cycle (SnPM test, p < 0.05)).

## Discussion

Compared to the conventional workflow, incorporating tuned muscle parameters when simulating muscle dynamics led to higher agreement between recorded and estimated values not used in the tuning step. Simulated muscle excitations of most muscles with workflow ALL closely resemble the on/off timing of EMGs in the literature. Incorporating tuned muscle passive force–length relationships substantially improves muscle excitations across the knee and hip joints compared to workflow LIN. Whole-body average metabolic rates were similar among workflows and in good agreement with measured values, i.e., mean differences between estimated and measured values varied between − 3.3 and 8.7%. Exact estimation was not expected, yet improvement in the estimation of fiber lengths and muscle excitations compared to workflow LIN and good agreement of metabolic rates compared to measured values lends confidence that the proposed tool can leverage prior experimental data in the literature to inform the computation of muscle–tendon parameters.

Tuned parameters obtained from optimizing fiber lengths agreed with reported values in the literature. Optimal fiber lengths were within one standard deviation from reported values from the cadaveric dataset from Ward et al.^25^. These estimated fiber lengths also agreed well with those obtained from MRI using diffusion tensor imaging^27^, except in soleus, where reported fiber lengths from Charles et al.^26^ were nearly double our estimated lengths as well as those reported by Ward et al.^25^. Interestingly, tuning parameters did not fully resemblance recorded fiber lengths in most of the muscles. Tuned parameters obtained by optimizing fiber lengths resulted in more isometric contractions in muscles informed by ultrasound imaging and improved the operating range of soleus compared to the workflow LIN. These improvements indicate that some features of the recorded fiber lengths were captured by tuning muscle–tendon properties. Nonetheless, fiber length excursion in gastrocnemius was larger than recorded fiber lengths, while in vasti, they were shorter. Disagreements in fiber excursion were still observed after adjusting the design variable bounds (Supplementary Fig. 6). Two simplifications in the musculoskeletal geometry might explain the persistent discrepancies. The musculoskeletal model proposed by Rajagopal et al. did not model the quadriceps tendon and patellar ligament separately, though they have different stiffnesses^28^. Fiber length excursion will be affected by tendon compliance as observed in the plantarflexors. Also, the foot is described as two segments—hind-/midfoot and toes—which might overestimate muscle–tendon lengths compared to multisegmented foot models^29^. Future studies that aim to represent muscle–tendon mechanics better might benefit from using our simulation workflow and incorporating a more detailed muscle–tendon geometry.

Tuned muscle passive force–length relationships resemble available experimental observations. Muscle passive force–length relationships of hamstrings, vasti, and gracilis have been reported to be lower, i.e., more compliant, than default values from the Thelen model^11,12^, which we also observed with our workflow ALL. In that regard, our optimization routine is suited to account for the variation of passive force–length relationships among muscles, as suggested in the literature. Computing passive moment–angle relationships requires recording the joint angles of a participant and the spatial location of hand-held load cells as well as a trained examiner who manipulates the limb through the range of motion passively in a standardized procedure^11,30^. In the absence of such equipment or personnel, researchers can use our tool to tune passive force–length relationships to resemble an in vivo pattern from the literature. Our work suggests that tuning such muscle properties is particularly relevant to accurately estimating muscle excitations and significantly influences metabolic rate trajectory.

Our proposed workflow (ALL) captured main muscle excitation features according to experimental values from Perry and other sources in the literature. As pointed out by Hick et al.^31^, it is recommended to compare estimated muscle activity, i.e., muscle excitations, with EMGs based on the on/off timing rather than its time-series trajectory directly—EMGs are challenging to normalize and subject to measurement error^32^. In this regard, we primarily evaluated our computed muscle excitations by comparing the on/off time with EMGs from Perry and, complementary, by computing the correlation coefficient. We selected the dataset from Perry since it consists of a comprehensive list of muscle excitations during preferred walking speed, and it agrees (to a large extent) with other datasets based on surface^33,34^ and fine-wire EMGs^35^. Surface EMGs allow measuring the activity of superficial muscles such as the soleus, gastrocnemius medialis and lateralis, tibialis anterior, peroneus longus and brevis, gluteus medius and maximus, tensor fasciae latae, rectus femoris, vastus medialis and lateralis, biceps femoris (long head) and semitendinosus^36^. The dataset provided by Perry agrees with other datasets based on surface EMGs except somewhat in rectus femoris^33,34^. In this muscle, surface EMGs are more sensitive to capturing crosstalk of nearby uniarticular knee extension muscles, and therefore, fine-wire EMGs are likely to be more reliable^37^. Prior studies using fine-wire EMGs estimated that rectus femoris is mainly active during the initial swing and not at the beginning and end of the gait cycle when walking near the preferred speed^35,37^, which is somewhat similar to our estimations. Deep muscles, on the other hand, cannot be measured with surface EMGs. Fewer experimental datasets are available for further comparison due to the invasive nature of recording muscle activities with fine-wire EMGs. Our estimated illiacus and psoas excitations, which were both primarily active during pre- and initial swing, disagree with values provided by Perry (psoas was not reported; thus, it was assumed the same as iliacus) but agree with the ones reported by Andersson et al.^35^. Other muscles where we did not estimate the on/off timing such as gracilis and piriformis (not available in Perry’s dataset) are relatively small compared to the total volume of the leg’s muscles (1.46 and 0.61%, respectively)^38^. Likely, not estimating the on/off timing of these muscles does not affect the results of validated outcomes, i.e., muscle excitations or metabolic rates.

Validation in muscle excitations and fiber lengths likely increases the confidence in the estimated metabolic rate trajectory. We cannot assert the accuracy of the estimated metabolic rate trajectories since they cannot be directly measured. Muscle metabolic energy consumption involves a series of slow cellular and systemic processes^39^; thus, instantaneous metabolic rates are typically approximated by recorded blood flow (in animal species)^40^ or computed muscle–tendon mechanics^41^. Despite the lack of recorded measurements, some features of the time-series metabolic cost are likely better represented with tuned parameters than simulation with the conventional workflow. Metabolic rates can be described as a function of muscle mechanical work, and heat quantities depend largely on muscle forces and fiber velocities, thus on muscle activation (and excitations) and fiber lengths^41^. In this regard, the closer agreement between recorded and estimated muscle excitations and fiber lengths using tuned parameters compared to workflow LIN increases the confidence in estimated metabolic rate trajectories. Tuning of parameters did not improve the accuracy of whole-body average metabolic rate estimations yet varied the metabolic rate estimations at the muscle group level, which might influence the estimated metabolic reduction using assistive devices^23^ or the relative cost of gait phases^42^.

Finally, our proposed approach has three inherent assumptions that warrant further discussion. First, fascicle lengths obtained from ultrasound imaging are representative measurements of the muscle length during contraction. Ultrasound imaging is a common modality to measure muscle contraction during dynamic contraction, such as walking^43^. While it is not exactly a “gold standard” due to its methodological limitations, it is a useful proxy to inform simulated muscle contraction. Second, passive force–length relationships are the same in muscles with multiple attachment points or that share similar functions. As previously stated, the estimated muscle force–length relationship resembles experimental observations in the hamstring, vasti, and gracilis. However, we cannot assert that our optimization provides realistic force–length relationships in the other muscles as we have not found reported experimental values in the literature. Third, optimal fiber length, tendon slack length, and tendon stiffness might be optimized separately from muscle passive force–length parameters. We did not optimize all parameters simultaneously but in sequence, assuming that our second optimization does not meaningfully influence the first. Our results support this assumption, as estimated fiber lengths were nearly unchanged with or without tuned muscle passive force–length relationships. Muscle parameters related to the active and passive force generation capacity can be optimized simultaneously if diverse sensor modalities, such as EMGs, dynamometer, and ultrasound imaging, are available^19–21^. Such comprehensive data collection, however, might be challenging to achieve due to the complexity of the experimental setups, which might confine it to describing single joint mechanics and a limited number of recorded muscle fibers. In the absence of a comprehensive dataset that includes recorded measurements in vivo from diverse sensor modalities, our methodology seems reasonable for decoupling and tuning muscle parameters.

## Conclusion

By tuning muscle–tendon parameters, simulations achieve more realistic estimations of fiber lengths, passive moment–angle relationships, and muscle excitations compared to a conventional workflow. Estimated whole-body average metabolic rates agree with recorded measurements regardless of the inclusion of tuned muscle–tendon parameters. Nevertheless, metabolic energy trajectory is sensitive to modeled muscle–tendon parameters, influencing metabolic rate estimation among muscle groups. This proposed tool could easily customize muscle–tendon parameters and is thus useful for improving simulated muscle–tendon mechanics. The code is freely available online at https://github.com/israelluis/Exoskeletons_ExperimentGuidedCalibration and can be easily adapted to incorporate fiber lengths of various muscles if such data is available.

## Methods

### Participants and experimental data

Twelve participants (7/5 male/female, [mean ± SD] age: 35.0 ± 9.1 years old, height: 1.76 ± 0.10 m, body mass: 74.9 ± 14.5 kg) participated in this experiment as part of a more comprehensive study to characterize energetics in gait. The study was approved by the Swedish Ethical Review Authority (Dnr. 2020-02311) in accordance with relevant guidelines and regulations, and all participants provided written informed consent. Participation was voluntary and could be terminated at any time during the experiment.

In the comprehensive study, participants walked from 55 to 145% of their preferred walking speed (PWS) during treadmill and overground walking. In this study, we used only motion data recorded at their PWS (1.46 ± 0.07 m/s [mean ± SD]). The PWS was estimated based on the participant’s gender, age, and height^44^. Participants walked at their PWS on a treadmill, and their cadence was recorded. They then walked overground along an instrumented walkway, and were asked to match their cadence from treadmill walking using audio signals from a metronome app. During treadmill walking, oxygen and carbon dioxide respiration rates were recorded for 6 min (Cortex Metamax 3B, Leipzig, Germany). The representative metabolic rate was computed based on the average in the last 3 min. During overground walking, marker position (100 Hz) and ground reaction forces (1000 Hz) were measured using optical motion capture (Vicon V16, Oxford, UK) and strain gauge force platforms (AMTI, Watertown, MA, USA), respectively. Full-body marker placement was implemented based on the Conventional Gait Model with the Extended-foot model (CGM 2.4)^45^. Ground reaction forces were processed using a 4th-order zero-lag Butterworth low-pass filter (35 Hz).

### Musculoskeletal model and inverse kinematics and dynamics

The generic musculoskeletal model proposed by Rajagopal et al.^46^ was selected for this study. We scaled the generic model using OpenSim’s Scale Tool, which adjusted muscle paths (origins/insertions), skeletal geometry, and segment inertial properties to fit anthropometric dimensions obtained from a captured static calibration trial and scaled the optimal fiber lengths and tendon slack lengths of each muscle–tendon actuator linearly to preserve the ratio of the generic model in the scaled model. The maximum isometric forces for each subject were computed from the expected muscle volume and specific tension (60 N/cm^2^)^46^. The expected muscle volume was calculated by Handsfield et al.^38^, which introduced a regression that estimates leg muscle volume based on subjects’ height and weight and muscle volumes as a percentage of the leg muscle volume.

Marker trajectories and ground reaction forces of one gait cycle were analyzed with inverse kinematics and inverse dynamics using OpenSim 4.1^7^. Marker tracking weights for inverse kinematics were selected to minimize errors between experimental and virtual markers in the musculoskeletal model. The subtalar and metatarsal joints were fixed at neutral anatomical positions. Joint kinematics and dynamics are reported in the Supplementary Material (Supplementary Fig. 7).

### Simulation workflow

**Figure 5:**
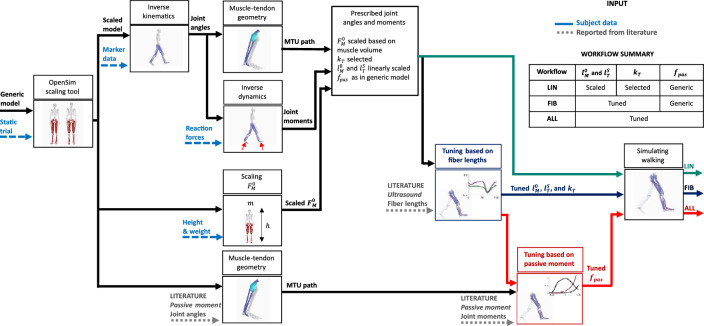
Simulation framework for each subject. Inverse kinematics and dynamics are computed using the OpenSim workflow. Moment arms and muscle–tendon lengths are computed from the inverse kinematic solution using the Muscle Analysis tool from OpenSim. We then proceed to tune parameters in the musculoskeletal model in steps. In the first step, optimal fiber length (${l}_{M}^{0}$), tendon slack length (${l}_{T}^{S}$) were tuned such that the computed muscle fiber lengths match reported findings during walking using ultrasound imaging.. In the second step, muscle passive force–length relationships (${f}_{pas}$) were tuned such that computed passive moments match the moment–angle joint relationship at various ankle, knee, and hip joint angles, reported in an in vivo study. Maximum isometric forces (${F}_{M}^{0}$) were scaled based on the expected muscle volume. We simulated muscle dynamics during walking using three workflows to analyze the influence of tuned parameters: muscle dynamics was computed using parameters from the conventional workflow (LIN), with tuned optimal fiber length, tendon slack lengths, and tendon compliance (FIB), and including tuned muscle passive force–length relationships (ALL).

We used trajectory optimization to simulate muscle dynamics^47^ and to optimize muscle–tendon parameters such that, for each subject, estimated fiber lengths and passive moment–angle relationships in the scaled musculoskeletal model better match in vivo experimental observations reported in the literature (Fig. 5). Our computational tool follows two steps: First, we optimized optimal fiber lengths, tendon slack lengths, and tendon stiffnesses to minimize the difference between recorded and estimated muscle fiber lengths. As a result of the first step, a set of tuned parameters was obtained for each muscle wherein recorded fiber length data was available. Then, using the tuned parameters, we optimized the muscle passive force–length relationships to minimize the difference between recorded and estimated joint passive moment–angle relationship. As a result of the second step, a set of tuned parameters is obtained for all the muscles in the musculoskeletal model (i.e., not only for muscles used to optimize the fiber lengths). The formulation of the optimization problem and experimental data for each step were detailed in the following sections. The proposed computational tool was implemented in Matlab R2020a (The Mathworks Inc., Natick, MA, USA).

### Optimization of parameters with ultrasound imaging

Estimated normalized fiber lengths in a subset of muscles of the musculoskeletal model were optimized to resemble recorded values using ultrasound imaging during motion. We optimized the normalized fiber lengths (rather than fiber lengths) as they directly relate to the muscles’ force-generation capacity. The normalized fiber lengths of six muscles were optimized: gastrocnemius lateralis and medialis, soleus, and vastus lateralis, medialis, and intermedius. These muscles were selected because their normalized fiber lengths during walking are available in (or can be inferred by) the literature.

We formulated an optimization routine to minimize the difference between recorded and estimated normalized fiber lengths while simulating muscle dynamics (Table 1). Muscle activation and contraction dynamics were modeled as in De Groote et al.^47^. Muscle redundancy was solved based on the minimization of an objective function, which consisted of four weighted terms: sum of squared muscle activations, differences between recorded and estimated normalized fiber lengths, sum of squared reserve actuators (i.e., a non-physiological ideal moment at each degree of freedom), and sum of squared muscle fiber velocities. The third term related to reserve actuators guaranteed the problem’s feasibility, and its weight was heavily penalized in the objective function (${w}_{r}=1000$). The fourth term was related to fiber velocities improved the numerical computation, and its weight was relatively small in the objective function (${w}_{v}=0.01$). Optimal fiber lengths, tendon slack lengths, and tendon stiffnesses of the six muscles previously mentioned were included as additional design variables. Two equilibrium conditions were imposed when simulating motion: the sum of muscle moments equaled the net joint moment at each degree of freedom, and muscle force equals tendon force at each muscle actuator. Joint angles and moments during simulated motion were prescribed—they are the same as those computed from the inverse kinematics and dynamics, respectively.

**Table 1:** Formulation of the optimization problems for each subject.

Optimization problem	Redundancy problem		
	Muscle forces informed by recorded fiber lengths	Passive muscle forces informed by recorded joint passive moment–angle relationship	Muscle forces
Objective function	$\text{min}\left({w}_{a}{\int }_{{t}_{i}}^{{t}_{f}}\sum_{n=1}^{N}{a}_{n}^{2}\left(t\right)dt+{w}_{f}{\int }_{{t}_{i}}^{{t}_{f}}\sum_{l=1}^{L}{\left({\widetilde{l}}_{{M}_{l}}\left(t\right)-{\widetilde{l}}_{{E}_{l}}\left(t\right)\right) }^{2}dt+{w}_{r}{\int }_{{t}_{i}}^{{t}_{f}}\sum_{j=1}^{J}{e}_{{R}_{j}}^{2}\left(t\right)dt+{w}_{v}{\int }_{{t}_{i}}^{{t}_{f}}\sum_{n=1}^{N}{\widetilde{v}}_{n}^{2}\left(t\right)dt\right)$ ${w}_{a}$=1, ${w}_{f}$=1, ${w}_{r}$=1000, and ${w}_{v}$=0.01	$\text{min}\left({w}_{p}\left(\frac{1}{N}\sum_{n=1}^{N}{\left(\frac{{k}_{p{e}_{n}}-{k}_{p{e}_{def} }}{100}\right)}^{2}+\frac{1}{N}\sum_{n=1}^{N}{\left(\frac{{s}_{{0}_{n}}-{s}_{{0}_{def} }}{100}\right)}^{2}+\frac{1}{N}\sum_{n=1}^{N}{\left(\frac{{s}_{{M}_{n}}-{s}_{{M}_{def} }}{100}\right)}^{2}\right) +{w}_{r}{\int }_{{t}_{i}}^{{t}_{f}}\sum_{j=1}^{J}{e}_{{R}_{j}}^{2}\left(t\right)dt\right)$ ${w}_{p}$=0.1 and ${w}_{r}$=1000	$$\text{min}\left(\begin{array}{c}{w}_{a}{\int }_{{t}_{i}}^{{t}_{f}}\sum_{n=1}^{N}{a}_{n}^{2}\left(t\right)dt\\ +{w}_{r}{\int }_{{t}_{i}}^{{t}_{f}}\sum_{j=1}^{J}{e}_{{R}_{j}}^{2}\left(t\right)dt\\ +{w}_{v}{\int }_{{t}_{i}}^{{t}_{f}}\sum_{n=1}^{N}{\widetilde{v}}_{n}^{2}\left(t\right)dt\end{array}\right)$$ ${w}_{a}$=1, ${w}_{r}$=1000, and ${w}_{v}$=0.01
Design variables	Muscle control, states, and state derivatives Tuning variables ${l}_{{M}_{l}}^{0},{l}_{{T}_{l}}^{s},{k}_{{T}_{l}}$ *for l* $=1,\dots ,L$	Joint passively moved, joint velocity assumed neglectable (${a}_{n},{\widetilde{v}}_{n}=0$) Tuning variables ${k}_{p{e}_{n}},{s}_{{0}_{n}},{s}_{{M}_{n}}$ *for n* $=1,\dots ,N$	Muscle control, states, and state derivatives
Constraints	Activation and contraction dynamics, as described by DeGroote et al.^47^ Muscles and net moment equilibrium ${\tau }_{I{D}_{j}}={\tau }_{{M}_{j}}+{e}_{{R}_{j}}{T}_{R}$ *for* $j=1,\dots ,J$ Muscle and tendon force equilibrium ${F}_{{M}_{n}}\text{cos}\left(\theta \right)={F}_{{T}_{n}}$ *for n* $=1,\dots ,N$ Non-normalized Achilles tendon ${k}_{{T}_{low}}^{non}\le \sum_{k=1}^{K}{k}_{{T}_{k}}^{non}\le {k}_{{T}_{high}}^{non}$	No activation or contraction dynamics Muscles and net moment equilibrium ${\tau }_{I{D}_{j}}={\tau }_{{M}_{j}}+{e}_{{R}_{j}}{T}_{R}$ *for* $j=1,\dots ,J$ Muscle and tendon force equilibrium ${F}_{{M}_{n}}\text{cos}\left(\theta \right)={F}_{{T}_{n}}$ *for n* $=1,\dots ,N$ ${k}_{p{e}_{n}},{s}_{{0}_{n}},{s}_{{M}_{n}}$ are constrained as in Luis et al	Activation and contraction dynamics, as described by DeGroote et al.^47^ Muscles and net moment equilibrium ${\tau }_{I{D}_{j}}={\tau }_{{M}_{j}}+{e}_{{R}_{j}}{T}_{R}$ *for* $j=1,\dots ,J$ Muscle and tendon force equilibrium ${F}_{{M}_{n}}\text{cos}\left(\theta \right)={F}_{{T}_{n}}$ *for n* $=1,\dots ,N$
Bounds	Muscle control, states, and state derivatives as in DeGroote et al.^47^ ${l}_{M}^{0}\in \left[{l}_{{M}_{LIN}}^{0}+/- 0.1*{l}_{{M}_{lin}}^{0}\right]$ ${l}_{T}^{s}\in \left[{l}_{{T}_{lin}}^{s}+/- 0.1*{l}_{{T}_{lin}}^{s}\right]$ ${k}_{T}\in \left[{k}_{{T}_{min}} {k}_{{T}_{max}}\right]$	Muscle control, states, and state derivatives as in DeGroote et al ${k}_{pe}\in [{k}_{p{e}_{def}}+/- 0.25*{k}_{p{e}_{def}}]$ ${s}_{0}\in [{s}_{{0}_{def}}+/- 0.25*{s}_{{0}_{def}}]$ ${s}_{M}\in [{s}_{{M}_{def}}+/- 0.33*{s}_{{M}_{def}}]$	Muscle control, states, and state derivatives as in DeGroote et al.^47^

Related to the states and objective function: $a$ is muscle activation, ${\widetilde{l}}_{M}$ is the normalized fiber length, and $\widetilde{v}$ is the normalized fibre velocity. ${t}_{f}$ and ${t}_{i}$ are the initial and final times of the gait cycle, respectively. ${w}_{a}$, ${w}_{f}$, ${w}_{r}$, ${w}_{v}$, and ${w}_{p}$ are the weight of the terms related to muscle activation, muscle fiber error, reserve actuator, and muscle passive parameter regularization, respectively.

Related to the optimized variables: ${\widetilde{l}}_{M}^{0}$ is the optimal fiber length, ${l}_{T}^{s}$ is the tendon slack length, ${k}_{T}$ is the normalized tendon stiffness, ${k}_{PE}$ is the exponential shape factor for the passive force–length relationship, ${s}_{0}$ is the normalized fiber length at which the passive force starts to increase, and ${s}_{M}$ is the normalized fiber length, measured from the optimal fiber length (${\widetilde{l}}_{M}=1$) at which maximum force is reached. In addition, ${k}_{T}^{non}$ is the non-normalized tendon stiffness as described by Stenroth et al. ^51^.

Related to the number of variables: $N$ is the total number of muscles in one leg of the musculoskeletal model, $L$ is the number of muscle fibers tracked, $K$ is the number of muscle fibers tracked in the plantarflexors, and $J$ is the number of joints in one leg of the musculoskeletal model.

Related to the constraints and bounds: ${\tau }_{I{D}_{j}}$ is the inverse dynamics joint moment, ${\tau }_{{M}_{j}}$ is the summed of all muscle moments, and ${e}_{{R}_{j}}$ is the excitation of the reserve actuator at the joint $j$, and $T$ is the maximum value of the reserve moment. ${l}_{{M}_{lin} }^{0}$ and ${l}_{{T}_{lin}}^{s}$ are the values of the optimal fiber length, and tendon slack length, respectively, computed from the OpenSim scaling tool (linear scaled to anthropometric dimensions). ${k}_{{T}_{min}}$ and ${k}_{{T}_{max}}$ are 10 and 35, respectively. ${k}_{T}$ for all the other muscles not optimized were 35. ${k}_{p{e}_{def}}$, ${s}_{{0}_{def}}$, and ${s}_{{M}_{def}}$ are 4,1, and 0.6, respectively. ${k}_{{T}_{low}}^{non}$ and ${k}_{{T}_{high}}^{non}$ are the bounds for the non-normalized Achilles tendon stiffness (sum of the soleus, gastrocnemius lateralis, and gastrocnemius medialis) assumed as 93 and 207 N/mm, respectively. They represented the minimum value minus one standard deviation (${k}_{{T}_{low}}^{non}$), and the maximum value plus a standard deviation (${k}_{{T}_{high}}^{non}$) of the values reported by Stenroth et al.^51^.

Regarding the recorded data, we digitalized the gastrocnemius lateralis, gastrocnemius medialis, and soleus fiber lengths reported by Farris and Raiteri^48^. They did not report optimal fiber lengths; thus, we normalized fiber length to its maximum value and multiplied by 0.9; these normalizations yield muscles to operate at the shallow ascending limb aligned with experimental observations^49^. We digitalized the vastus lateralis fibers reported by Bohm et al.^50^ and normalized them by the optimal fiber length reported in their study. We assumed the vastus medialis and intermedius to operate in the same region as the vastus lateralis.

Tuned parameters in this step were optimized simultaneously using motion data (computed joint angle and moments) of one gait cycle. Therefore, we obtained a set of tuned parameters for each subject of our study.

### Optimization of parameters with moment–angle relationship

Estimated joint passive moment–angle relationships at various ankle, knee, and hip angles were optimized to resemble recorded values obtained from motion data and hand-held 3D load cells. We used published data from Silder et al.^30^, digitalized by Uhlrich et al.^52^. Dataset of Silder et al. was selected as it provided average values from a comprehensive sample population (i.e., twenty healthy young adults) at distinct joint configurations in the sagittal plane: passive ankle moments across its range of motion at 0°, 15°, and 60° knee flexion; passive knee moments across its range of motion at 20° dorsiflexion and hip neutral position, 15° plantarflexion and hip neutral position, and 20° dorsiflexion and 15° hip extension; and passive hip moments across its range of motion at 15° and 60° knee flexion. Passive force–length relationships in all muscles were optimized using as input the joint passive moment–angle relationships and prior tuned parameters obtained from optimized fiber lengths (described in the previous section).

We formulated an optimization routine to minimize the difference between recorded and estimated joint passive moment–angle relationship (Table 1). Muscle forces were computed assuming muscle activations and muscle velocities as zero, i.e., no activation and contraction dynamics were modelled, and, therefore, muscles only produced forces passively. Muscle passive force–length relationship was modeled based on Thelen^53^, as in Eq. (1)

1 $${f}_{pas}\left({\widetilde{l}}_{M}\right)=\frac{{e}^{\frac{{k}_{PE}\left({\widetilde{l}}_{M}-{s}_{0}\right)}{{s}_{M}}}-1 }{{e}^{{k}_{PE}}-1}$$

where ${\widetilde{l}}_{M}$ is the normalized fiber length, ${k}_{PE}$ is the exponential shape factor for the passive force–length relationship, ${s}_{0}$ is the normalized fiber length at which the passive force starts to increase, and ${s}_{M}$ is the normalized fiber length, measured from the optimal fiber length (${\widetilde{l}}_{M}=1$), at which maximum force is reached.

Muscle (passive force) redundancy was solved based on the minimization of an objective function, which consisted of four weighted terms: three terms related to penalizing the squared differences between tuned and generic passive force–length parameters values, and one related to the sum of squared reserve actuators. The ${k}_{PE}$, ${s}_{0}$, and ${s}_{M}$ of all muscles were included as additional design variables. Two equilibrium conditions were imposed when simulating motion: the sum of muscle moments equaled the net joint moment at each degree of freedom, and muscle force equals tendon force at each muscle actuator. Joint angles and moments during simulated motion were as in Silder et al.^54^.

Additional considerations were taken in this optimization routine to ensure convergence. We assumed that the muscle passive parameters were the same within muscles with multiple attachment points or that share similar functions, as in Luis et al.^14^, to reduce the number of design variables. We slightly adjusted the tendon slack length of the semimembranosus. After using the OpenSim scaling tool, this muscle was too short in positions with hip flexion and full knee extension, which resulted in negative normalized fiber length at those joint configurations. Therefore, the semimembranosus’s tendon slack length was recomputed so that the semimembranosus fiber length was non-negative in this step.

Tuned parameters in this step were optimized using all the passive moment–angle relationships of the lower limbs simultaneously, i.e., only one optimization problem was formulated where passive ankle, knee, and hip moment across their range of motion in distinct joint configurations were inputs and tuned parameters related to muscle passive force–length relationships were outputs. We solved for all joints simultaneously as some muscles generate passive force spanning multiple joints, e.g., gastrocnemius, and, thus, solving force redundancy cannot be solved at each joint independently. As a result of this step, we obtained a set of tuned parameters for each subject of our study.

### Experimental and estimated metabolic rates

Experimental and estimated whole-body average metabolic rates were computed from recorded measurements and simulation workflows, respectively. The experimental whole-body average metabolic rate was computed based on the representative (3 min averaged) oxygen and carbon dioxide rate values using the Brockway equation^55^. The estimated whole-body average metabolic rate was computed based on the metabolic energy model proposed by Bhargava et al.^41^. In brief, muscle control/excitation, states, and state derivatives are used to compute the metabolic rates of each muscle (${\dot{E}}_{n}$) based on muscle work rate and heat dissipation rate. Detailed information about such computation can be found in the respective article^41^. Additionally, we adjusted the heat dissipation rate to constrain muscle metabolic rate to be non-negative as implemented by Uchida et al.^42^ This metabolic energy model was selected because it yields closer agreement with experimental observations obtained from spirometry compared to other models in literature^14^. The metabolic rate trajectory of one leg (${\dot{E}}_{leg}$) was computed as the sum of all the muscle metabolic rates simulated during a gait cycle as in Eq. (2):

2 $${\dot{E}}_{leg}(t)=\sum_{n=1}^{N}{\dot{E}}_{n}(t)$$

The estimated net whole-body average metabolic rate was calculated as the mean value of the time-series metabolic rates of one leg and multiplied by two to account for two legs (as we simulated the dynamics of one leg). The estimated (gross) whole-body average was calculated as the net whole-body average plus a basal rate assumed to be 1.2 W/kg^56^.

### Data and statistical analysis

We used the same simulation framework to perform three workflows: Workflow LIN uses the conventional linear scaling workflow in OpenSim; workflow FIB uses tuned optimal fiber length, tendon slack length, and tendon stiffness; and finally, workflow ALL uses the previously optimized parameters and also tuned the muscle passive force–length relationships. In workflow LIN, we selected the non-dimensional tendon stiffness (${k}_{T}$) as 15 for Achilles tendon. This assumption allows for a fairer comparison between typically used values, which assume such tendons as highly compliant^57,58^ and our workflow. As a reference, this stiffness value yields a non-normalized Achilles tendon of 150 N/mm in the generic model, which aligns with experimental observations ^51^. We compared non-normalized Achilles tendon values using the generic tendon stiffness and the one tuned by optimizing fiber lengths using the Wilcoxon signed-rank test (n = 12). This statistical method was selected due to the non-normal data distribution and dependency. A significant level of 0.05 was chosen to determine statistical significance, and the null hypothesis tested was that there were no significant differences between compared groups.

The agreement between recorded and estimated fiber lengths and passive moment–angle relationships among workflows was examined. This comparison served to evaluate the degree to which tuning parameters resemblance experimental data. We computed the Pearson’s linear correlation coefficient (*r*) and root-mean-square error (RMSE) between the experimental fiber lengths (used in parameter tuning) and estimated fiber lengths from each workflow and between the experimental passive moment–angle relationship (used in parameter tuning) and workflows FIB and ALL. Also, the influence of tuning parameters in the estimated fiber velocities and forces was examined.

The agreement between experimental and estimated muscle excitations and metabolic rates among workflows was examined. This comparison served to evaluate the degree to which tuning parameters better estimate quantities not included in the tuning process. We compared the on/off timing qualitatively and computed the correlation coefficient between the estimated muscle excitations in each workflow with experimental findings from Perry^59^. This author presented a comprehensive dataset of recorded muscle activity using fine-wire EMGs during walking in various superficial and deep muscles in non-disabled individuals. It was assumed that adductor brevis, psoas, and gluteus minimus (which were not reported in Perry’s dataset) have the same muscle excitations as the adductor longus, iliacus, and gluteus medius, respectively, as they can exert force within the same muscle function groups. Also, piriformis was not evaluated as no EMGs were reported^59^. Related to metabolic energy cost, we used a Wilcoxon signed-rank test (n = 12) with a significant level of 0.05 to evaluate the difference between the experimental and estimated whole-body average metabolic rates for each workflow. We compared the metabolic rate trajectories of one leg (${\dot{E}}_{leg}$) between workflow LIN and FIB, workflow FIB and ALL, and workflow LIN and ALL using a one-dimensional statistical non-parametric mapping toolbox^60,61^. This statistical technique compared time frames through a statistical non-parametric mapping (SnPM, alpha = 0.05, two-tailed paired t-test)^61^. This approach has served to identify statistically significant differences between computational frameworks within gait phases^16^. All the statistical analyses were conducted using Matlab R2023b (The Mathworks Inc., Natick, MA, USA).

### Supplementary Information

Supplementary Information.

## Data Availability

Requests for dataset used and analyzed during the current study are available from the corresponding author I. L. on reasonable request. The code used in this study is freely available online at https://github.com/israelluis/Exoskeletons_ExperimentGuidedCalibration
