# Supplementary material for: Experiment-guided tuning of muscle–tendon parameters to estimate muscle fiber lengths and passive forces
Source: Sci Rep. 2024 Jun 25;14:14652. doi: 10.1038/s41598-024-65183-1 (PMC11199655; doi:10.1038/s41598-024-65183-1)
Supplement: Supplementary file 1 — Supplementary Information. [file 41598_2024_65183_MOESM1_ESM.docx]

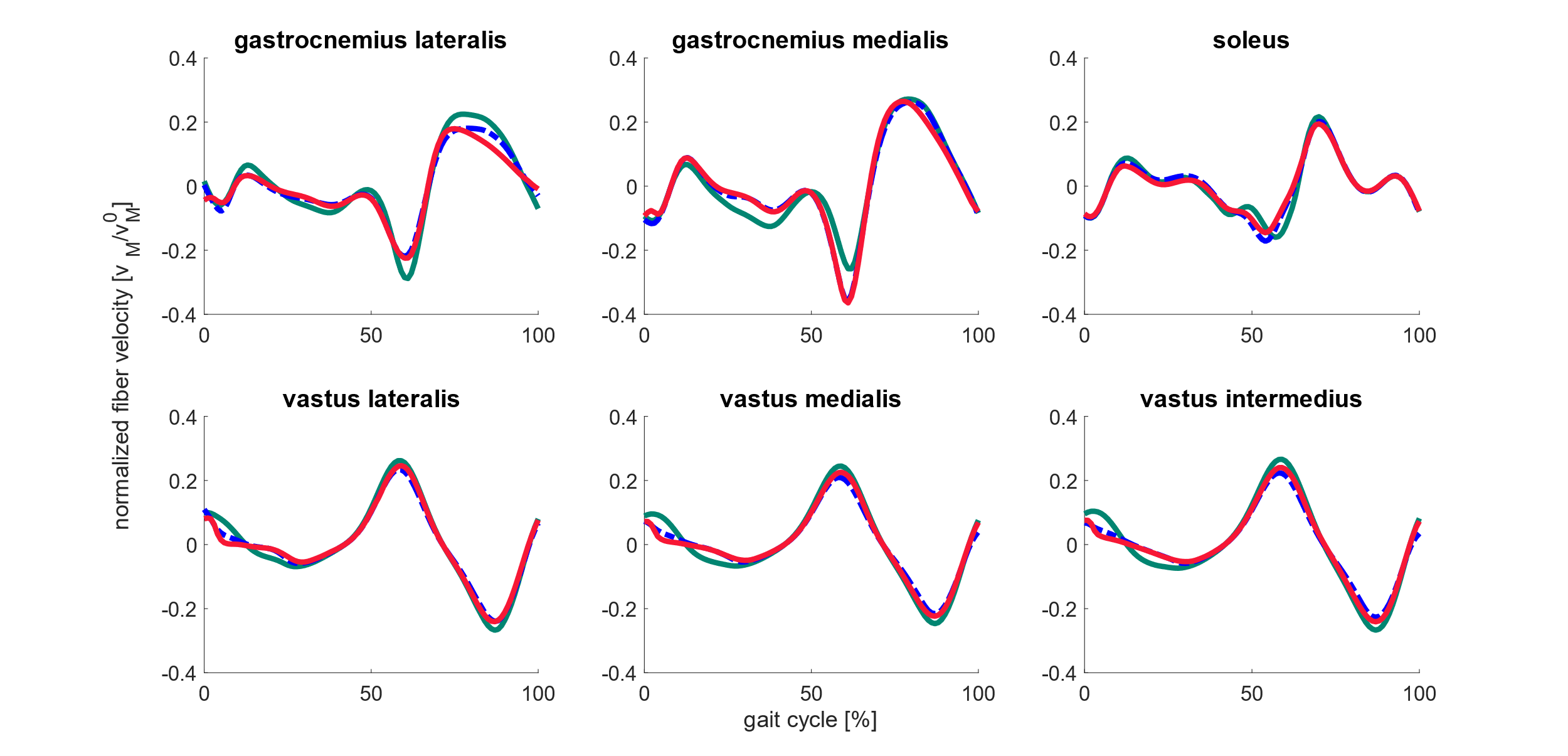


Supplementary Figure 1. Computed fiber velocities. Average fiber velocities computed from the conventional workflow (LIN, *green*), with tuned optimal fiber length, tendon slack lengths, and tendon compliance (FIB, *dashed* *blue*), and including tuned muscle passive force-length relationships (ALL, *red*). Average values are computed among all subjects.


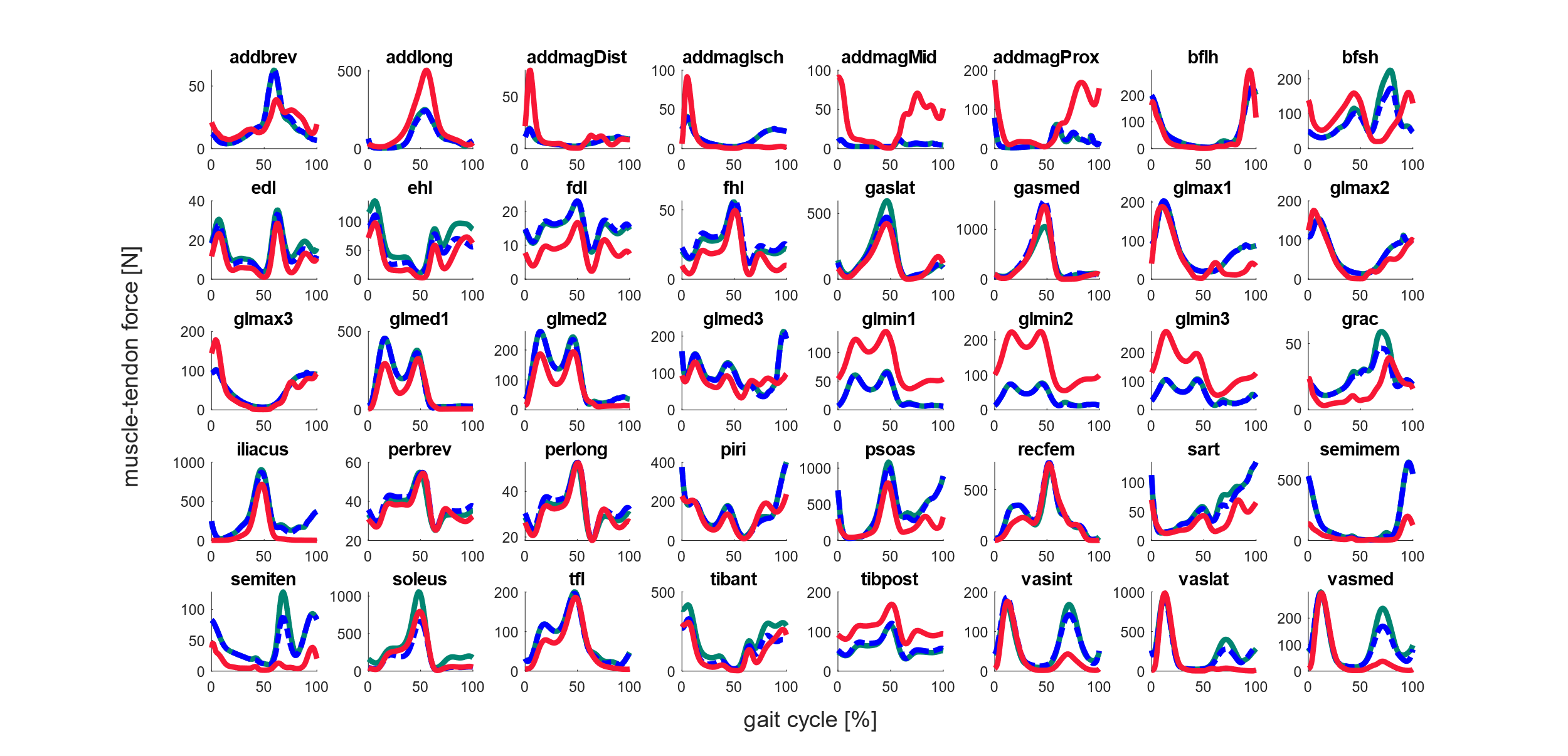


Supplementary Figure 2. Computed muscle-tendon forces. Average muscle-tendon forces computed from the conventional workflow (LIN, *green*), with tuned optimal fiber length, tendon slack lengths, and tendon compliance (FIB, *dashed* *blue*), and including tuned muscle passive force-length relationships (ALL, *red*). Average values are computed among all subjects.


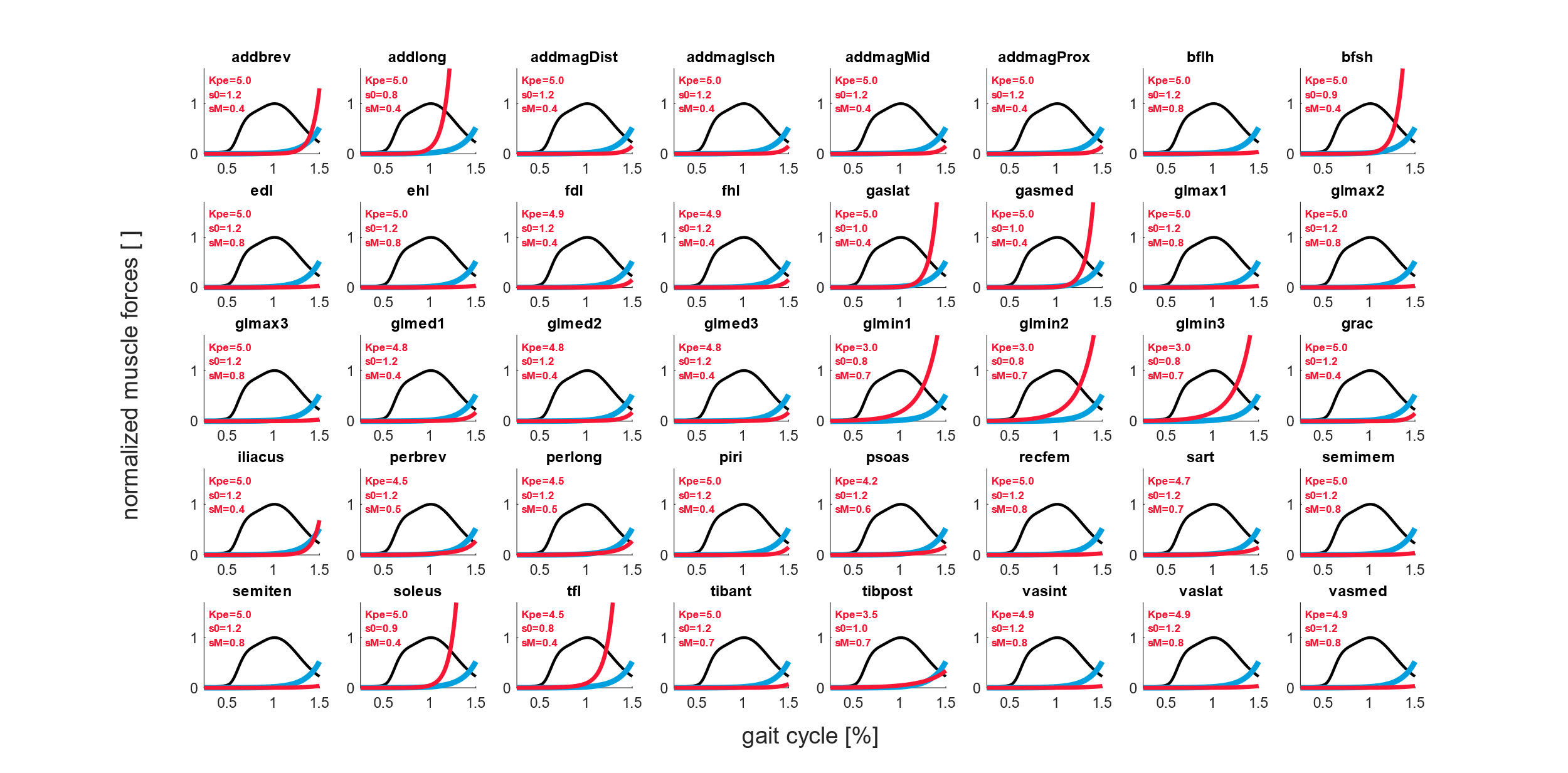


Supplementary Figure 3. Active and passive force-length relationship in muscles. Average muscle passive force-length relationships with generic (*light blue*) and tuned (*red*) parameters. Muscle names (plot titles) refer to their abbreviations in the musculoskeletal model: adductor brevis (addbrev), adductor longus (addlong), adductor magnus (addmagDist, addmagIsch, addmagMid, and addmagProx), biceps femoris long head (bflh), biceps femoris short head (bfsh), extensor digitorum longus (edl), extensor hallucis longus (ehl), flexor digitorum longus (fdl), flexor hallucis longus (fhl), gastrocnemius lateralis (gaslat), gastrocnemius medialis (gasmed), gluteus maximus (glmax1, glmax2, and glmax3), gluteus medialis (glmed1, glmed2, and glmed3), gluteus minimus (glmin1, glmin2, and glmin3), gracilis (grac), iliacus, peroneus brevis (perbrev), peroneus longus (perlong), piri, psoas, rectus femoris (recfem), sartorius (sart), semimembranosus (semimem), semitendinosus (semiten), soleus, tensor fasciae latae (tfl), tibialis anterior (tibant), tibialis posterior (tibpost), vastus intermedius (vasint), vastus lateralis (vaslat), and vastus medialis (vasmed). Generic values for $k_{PE}$, $s_{0}$, and $s_{M}$ are 4, 1, and 0.6, respectively. Average values among all the subjects are illustrated.


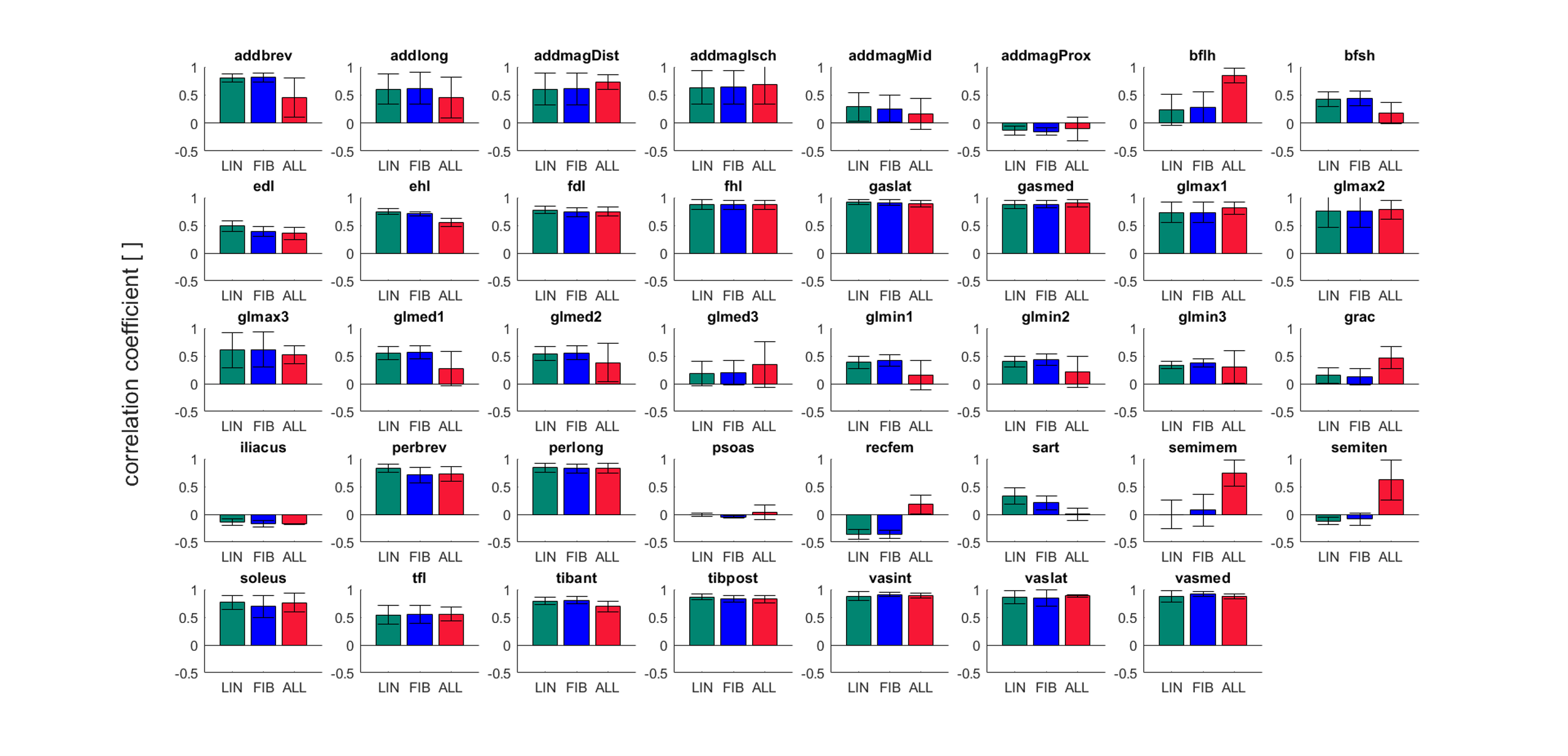


Supplementary Figure 4. Correlation coefficients between experimental and computed muscle excitations. Correlation coefficients between EMGs and muscle excitations computed from the conventional workflow (LIN, *green*), with tuned optimal fiber length, tendon slack lengths, and tendon compliance (FIB, *blue*), and including tuned muscle passive force-length relationships (PAS, *red*). EMG signals were obtained by digitalizing data reported by Perry^41^. Muscle names (plot titles) refer to their abbreviations in the musculoskeletal model: adductor brevis (addbrev), adductor longus (addlong), adductor magnus (addmagDist, addmagIsch, addmagMid, and addmagProx), biceps femoris long head (bflh), biceps femoris short head (bfsh), extensor digitorum longus (edl), extensor hallucis longus (ehl), flexor digitorum longus (fdl), flexor hallucis longus (fhl), gastrocnemius lateralis (gaslat), gastrocnemius medialis (gasmed), gluteus maximus (glmax1, glmax2, and glmax3), gluteus medialis (glmed1, glmed2, and glmed3), gluteus minimus (glmin1, glmin2, and glmin3), gracilis (grac), iliacus, peroneus brevis (perbrev), peroneus longus (perlong), piri, psoas, rectus femoris (recfem), sartorius (sart), semimembranosus (semimem), semitendinosus (semiten), soleus, tensor fasciae latae (tfl), tibialis anterior (tibant), tibialis posterior (tibpost), vastus intermedius (vasint), vastus lateralis (vaslat), and vastus medialis (vasmed). Mean values and one standard deviation among all subjects are illustrated.


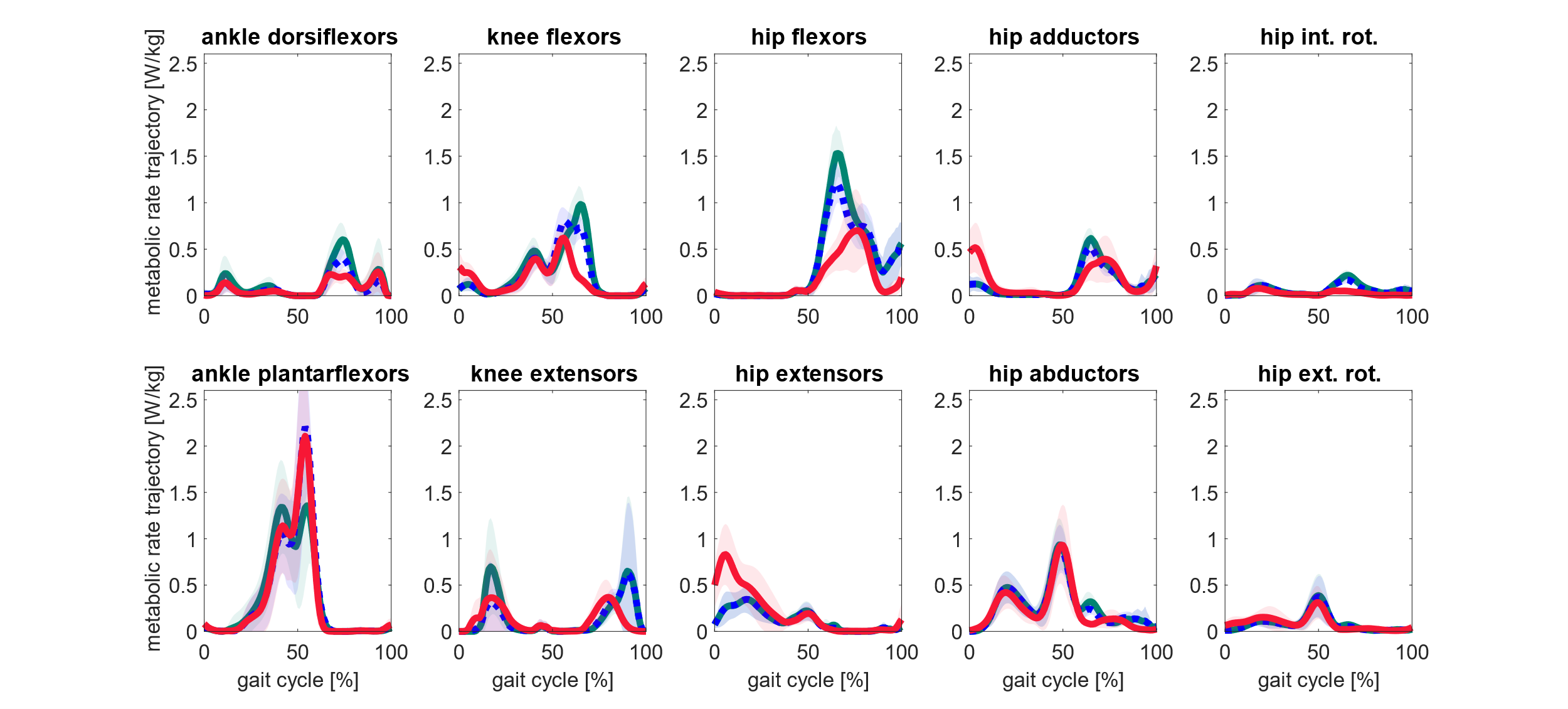


Supplementary Figure 5. Computed metabolic rate trajectories of muscle groups. Average metabolic rate trajectories of the lower-limb muscle groups computed from the conventional workflow (LIN, *green*), with tuned optimal fiber length, tendon slack lengths, and tendon compliance (FIB, *dashed* *blue*), and including tuned muscle passive force-length relationships (ALL, *red*). Average values are computed among all subjects, scaled by their mass.


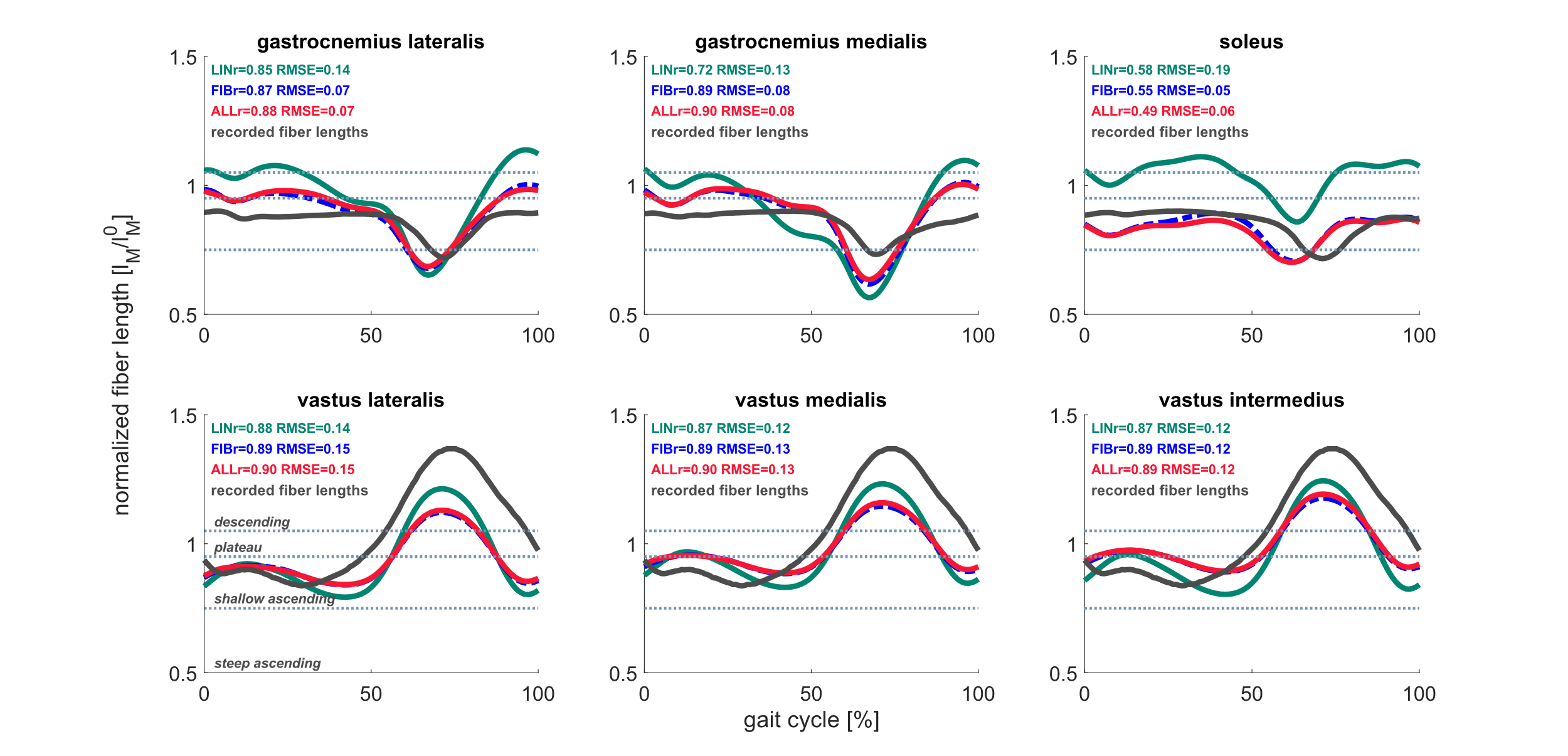


Supplementary Figure 6. Computed and experimental fiber lengths based on parameters with larger bounds. Average fiber lengths computed from the conventional workflow (LIN, green), with tuned optimal fiber length, tendon slack lengths, and tendon compliance (FIB, *dashed blue*), and including tuned muscle passive force-length relationships (ALL, *red*). Optimal fiber lengths and tendon slack lengths were bounded +/- 50% of their original value.


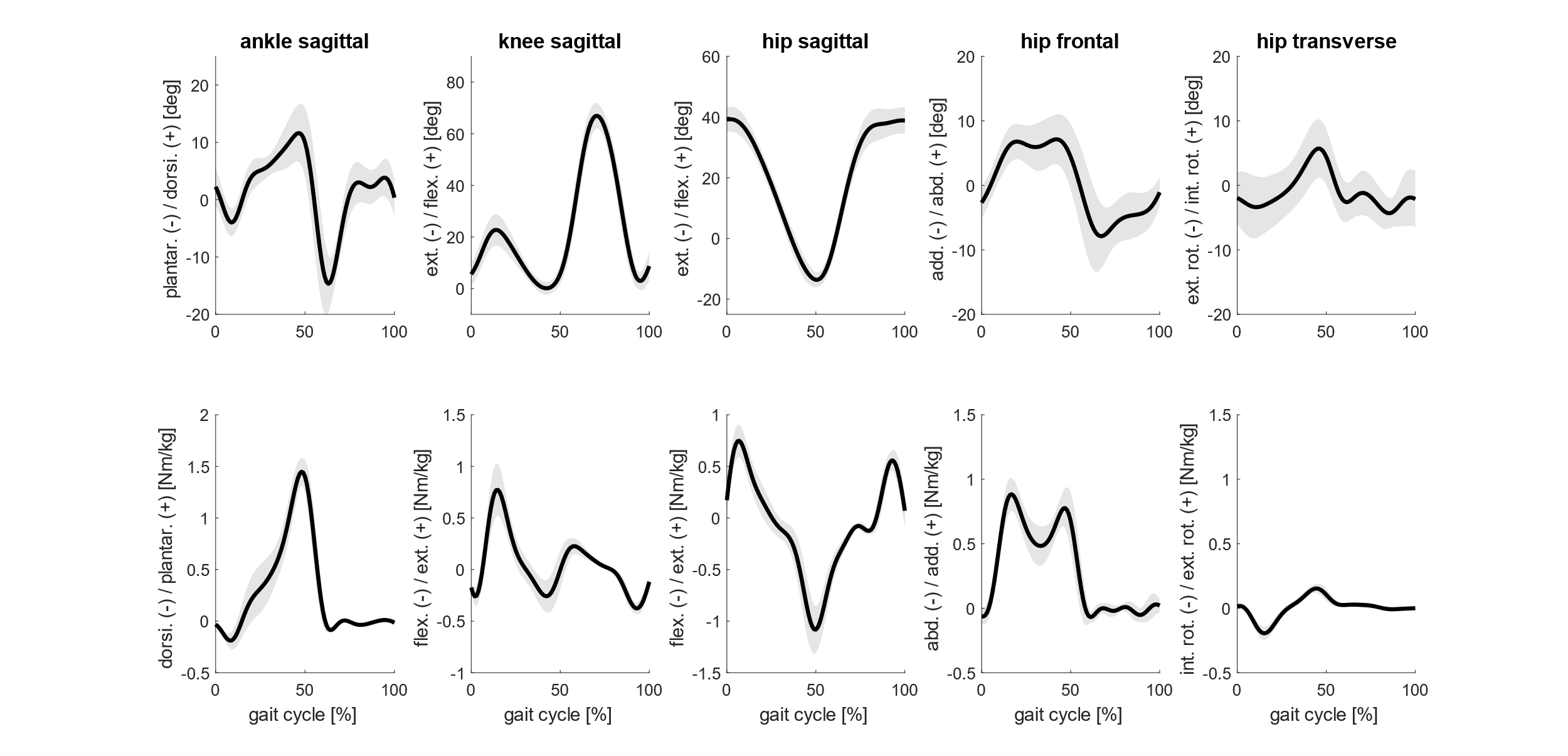


Supplementary Figure 7. Joint angles and moments of the lower limbs. Joint angles (upper row) and joint moments (lower row) represented the average values among all subjects. Joint moments were scaled by their subject’s mass.

Supplementary Table 1. Computed and experimental optimal fiber lengths. Mean and one standard deviation (MEAN [STD]) of optimal fiber lengths with the conventional workflow (LIN), tuned parameters based on optimized fiber lengths (TUNED), the cadaveric dataset from Ward et al., and MRI dataset from Charles et al. Values from the cadaveric dataset were obtained from the respective publication, while values from the MRI dataset were computed based on ten subjects reported in Charles et al.

| Muscles | Optimal fiber lengths | | | |
| --- | --- | --- | --- | --- |
|  | LIN | TUNED | CADAVERIC | MRI |
| Gastrocnemius lateralis | 6.10 [0.43] | 6.71 [0.47] | 5.80 [1.00] | 5.86 [1.52] |
| Gastrocnemius medialis | 5.30 [0.37] | 5.82 [0.40] | 5.10 [1.00] | 6.15 [1.78] |
| Soleus | 4.59 [0.32] | 5.05 [0.36] | 4.40 [1.00] | 13.46 [2.92] |
| Vastus lateralis | 9.91 [0.46] | 10.90 [0.50] | 9.90 [1.80] | 9.88 [2.05] |
| Vastus medialis | 9.60 [0.45] | 10.56 [0.50] | 9.70 [2.30] | 10.78 [2.52] |
| Vastus intermedius | 9.85 [0.47] | 10.84 [0.51] | 9.90 [2.00] | 10.17 [2.35] |
